# Opportunities to improve open All of Us data to convey CYP2D6 pharmacological relevance and interpretation overall and according to ancestry

**DOI:** 10.3389/fphar.2026.1760362

**Published:** 2026-04-13

**Authors:** Rachele M. Hendricks-Sturrup, Kurt D. Christensen, Christine Y. Lu

**Affiliations:** 1 Duke-Robert J. Margolis Institute for Health Policy, Duke University, Washington, DC, United States; 2 Department of Population Medicine, Harvard Pilgrim Healthcare Institute, Boston, MA, United States; 3 Department of Population Medicine, Harvard Medical School, Boston, MA, United States; 4 Kolling Institute, Faculty of Medicine and Health, The University of Sydney and the Northern Sydney Local Health District, Sydney, NSW, Australia; 5 School of Pharmacy, Faculty of Medicine and Health, The University of Sydney, Sydney, NSW, Australia

**Keywords:** ancestry, CYP2D6, genomic medicine, pharmacogenomics, return of results, bioinformatics, open data

## Abstract

**Introduction:**

*CYP2D6* is a highly polymorphic gene with significant pharmacogenomic and public health implications. Understanding the population distribution of *CYP2D6* variants offers opportunities to enhance awareness of its pharmacological relevance, particularly across different ancestral groups.

**Methods:**

Using the National Institute of Health *All of Us* Research Program Data Browser, we identified *CYP2D6* alleles with a frequency ≥0.10 in the overall cohort or within ancestral subgroups and classified in ClinVar as having either “drug response” or “undefined” significance. We assessed alleles with frequency differences ≥0.10 between ancestral subgroups and the overall population to highlight subgroup-specific pharmacogenomic relevance. *CYP2D6* alleles with ClinVar “undefined” significance classifications were cross-referenced with expert pharmacological resources [e.g., Pharmacogene Variation (PharmVar) Consortium] to identify candidates for reclassification as “drug response” significance variants.

**Results:**

Among the 89 *CYP2D6* alleles with ClinVar “drug response” significance classification, 7 of 11 (64%) were more frequent (≥0.10) in the East Asian ancestry subgroup than in the overall population. Among 6,262 *CYP2D6* alleles with “undefined” significance classification, 60 showed elevated frequencies (≥0.10) in at least one subgroup: Middle Eastern (31/60; 52%), African (22/60; 37%), East Asian (21/60; 35%), and South Asian (4/60; 7%) ancestries. Of these 60 alleles, 16 (27%) map to 41–76 star allele haplotypes that contain at least one sub-allele with “definitive” allele evidence levels in PharmVar.

**Conclusion:**

Our findings highlight the differential distribution and potential pharmacological relevance of *CYP2D6* alleles across ancestral subgroups. These insights support potential reclassification of certain “undefined” alleles and highlight the importance of inclusive pharmacogenomic research to improve population-specific drug response knowledge.

## Introduction

The *CYP2D6* gene encodes human cytochrome P450 2D6, an enzyme heavily implicated in drug metabolism and highly homologous to its neighboring pseudogenes *CYP2D7* and *CYP2D8* (Rowland et al., 2006; Bank, RCSB Protein Data, 2026; Pratt et al., 2021). Recognized as a very important pharmacogene (VIP) by ClinPGx, an affiliate grant of the Clinical Genome Resource that integrates PharmGKB, CPIC, and PharmCAT projects, *CYP2D6* plays a central role in pharmacogenomics. Current databases list 175 instances of prescribing information, 119 clinical annotations, and implications in 68 therapeutic pathways, highlighting its broad clinical significance (ClinPGx, 2025). At present, the United States (US) Food and Drug Administration (FDA) has approved 72 labeling indications across 13 therapeutic areas for *CYP2D6* as a pharmacogenomic biomarker (see in Supplementary Table 1). Altogether, these developments indicate that evidence concerning the phenotypic importance of *CYP2D6*, as a VIP and FDA-approved pharmacogenomic biomarker, is currently or becoming increasingly sufficient for clinical implementation.

However, clinical implementation of *CYP2D6* testing remains uneven in practice, partially due to a current lack of consensus on the clinical utility of pharmacogenomic testing for *CYP2D6*, further complicating its uptake in routine care. For example, the Royal College of Pathologists of Australasia states that *CYP2D6* pharmacogenomic testing should be considered when prescribing amitriptyline, atomoxetine, codeine, nortriptyline, and tramadol (The Royal College of Pathologists of Australasia, 2025). Joint consensus recommendations among professional molecular pathology societies are helpful in addressing inconsistent allele classifications that persist across clinical molecular testing contexts for *CYP2D6* (Pratt et al., 2021; Kane, 2025; Chan et al., 2022).

The return of *CYP2D6* test results in national research settings, as opposed to clinical contexts, faces similar challenges. For example, the US National Institute of Health (NIH) *All of Us* Research Program (“*All of Us”*), a national research initiative with the mission to generate and return genetic test results to one million demographically diverse participants, currently excludes *CYP2D6* from its personalized medication reports due to the complexity of *CYP2D6* phenotype determination, which depends on both structural and copy number variants (NIH All of Us Research Program, 2020). Although *All of Us* has expressed plans to return *CYP2D6* results in the future, this will occur once there is greater clarity and consensus regarding the interpretation of these complex variants and standardization in how such interpretations are communicated (NIH All of Us Research Program, 2020).

Although personalized reports on *CYP2D6* status are not yet provided to *All of Us* participants, the *All of Us* Data Browser (http://databrowser.researchallofus.org/snvindel-variants) provides aggregate information concerning *CYP2D6* variants across the cohort. Aggregate genomic data are structured to reference single-nucleotide polymorphism (SNP) or ‘rs’ number information. These data can be stratified by genetic ancestry and the US National Library of Medicine ClinVar significance level. These convey the presence and type of variants found among *All of Us* participants.

*CYP2D6* is highly polymorphic, with more than 130 star allele haplotypes, and there has recently been a lack of standards to describe copy number and structural variation in *CYP2D6*. Moreover, recent studies show that *CYP2D6* variants can vary greatly by genetic ancestry, reflecting interindividual, ethnic, and genetic differences in *CYP2D6*-mediated drug metabolism (Pratt et al., 2021; Ozawa et al., 2004; Céspedes-Garro et al., 2014; Friedrich et al., 2014; Gaedigk et al., 2017; Rodrigues-Soares et al., 2020; Bradford and Kirlin, 1998; Kehinde et al., 2023). In this context, the publicly available *All of Us* data may serve as a helpful and accessible resource for lay users, including patients and patient caregivers, to obtain objective and trustworthy genomic information related to their health, and for scientific users seeking to formulate hypotheses regarding genetic ancestry and *CYP2D6* ancestry-based genotype and phenotype stratification. To date, however, no study has summarized *CYP2D6* data available within the publicly accessible Data Browser or evaluated its potential to support evidence-based clinical and translational research focused on validating or further exploring the pharmacological relevance of *CYP2D6* through the lens of human ancestry.

In this study, we describe our process to address this gap and anticipate that this work may be useful in two ways: first, to explore using the variety and ancestry prevalence of *CYP2D6* alleles with known or undefined pharmacological significance within the *All of Us* cohort, and second, to identify opportunities to improve public knowledge about the pharmacological relevance of *CYP2D6* open data platforms such as the *All of Us* Data Browser. We believe that such work is necessary to inform, support, and potentially inspire public and community engagement, as well as interest in the transparent interpretation and objective evaluation of genomic findings that could impact health and care management.

## Methods

### Data collection and assessment

A search was conducted in October 2024 within the *All of Us* Data Browser, which, since February 2023, has contained data from 409,420 participants in the United States (see *All of Us* Research Program Protocol in Supplement 2). The *All of Us* Data Browser contains summary-level statistics to ensure the privacy of individual-level information while preserving the relevance, reliability, and quality of *All of Us* data (NIH All of Us Research Program, 2025). The search targeted all single-nucleotide variants and short insertions/deletions (i.e., SNV/indel variants), using “*CYP2D6*” as the sole search term to determine the frequencies of *CYP2D6* variants labeled with “drug response” or “undefined” significance. Search results were recorded in Microsoft Excel and analyzed descriptively among three authors (RH-S, CYL, and KC) to assess ClinVar status per gene variant (“drug response” significance or “undefined” significance classifications, whereas “undefined” exists as a default value for unassigned variants) (National Center for Biotechnology Information, 2017).

Our analyses centered on *CYP2D6* alleles that met the following two criteria: a frequency difference of ≥0.10 compared with the overall population and a “drug response” or “undefined” significance classification in ClinVar (in alignment with the *All of Us* Data Browser convention). We further considered alleles with subgroup frequencies ≥0.10 that exceeded their frequency in the overall population. Data were further stratified by subgroup ancestry to 1) examine how knowledge of even relatively common *CYP2D6* variants might differ by subgroup (reported in aggregate form within the Data Browser) and 2) identify *CYP2D6* alleles that could warrant further pharmacological and evidence-based clinical practice considerations based on allele status in the Pharmacogene Variation (PharmVar) Consortium (https://pharmvar.org/) as of July 2025.

### Data summarization

Five data elements/variables from the All of Us Data Browser were recorded for *CYP2D6* variants: ClinVar annotations as “drug response” or “undefined” significance, variant ID, rs number, allele frequency, and ancestry. Variants stratified by participant ancestry were derived from whole-genome sequencing, single-nucleotide polymorphisms (SNPs), and insertions/deletions (indels). Single-variant ID and allele frequencies were recorded and calculated overall and across seven (7) ancestral categories: African, East Asian, European, Latin American (Americas; American Admixed/Latino), Middle Eastern, South Asian, and Other (Remaining; not belonging to one of the other ancestries or is a balanced admixture) (NIH All of Us Research Program, 2025). Native American and Alaska Native populations are not included in the available ancestry categories due to tribal sovereignty considerations and ongoing consultation between tribal nations and the All of Us Research Program (NIH All of Us Research Program, 2023). Recorded variant IDs and rs numbers for variants with ClinVar “undefined” significance were compared to or cross-referenced with relevant information in PharmVar (https://www.pharmvar.org/gene/ *CYP2D6*) to identify *CYP2D6* alleles that would warrant reclassification to ClinVar “drug response” significance. Tables were generated in Microsoft Excel to assess and summarize findings. The “STrengthening the Reporting Of Pharmacogenetic Studies” (STROPS) guideline (www.strops-guideline.org), along with published NIH recommendations for reporting genetic results, was used to align our work with current best practices on the reporting of pharmacogenetic studies and results (Chaplin et al., 2020; Bookman et al., 2006).

## Results

### Overall population summary

Up to February 2023, a total of n = 6,359 *CYP2D6* variants were observed among 245,460 participants with short-read whole-genome sequencing results within the *All of Us* database/cohort (overall study population; called against the GRCh38/hg38 genome reference). Among the overall *All of Us* study population, 53.3% (n = 145,580) reported female, 38.6% (n = 94,760) reported male, and 2.1% (n = 5,080) reported other sex assigned at birth. Self-reported race/ethnicity within the Data Browser showed the following: Asian (3.0%; n = 7,440); Black, African American, or African (20.4%; n = 50,080); Hispanic, Latino, or Spanish (17.1%; n = 41,940); White (51.3%; n = 125,860); more than one race/ethnicity (3.8%; n = 9,220); other (1.7%; n = 4,040); and prefer not to answer (2.8%; n = 6,880).

Among *All of Us* participants with reported *CYP2D6* genotypes, 1.4% (n = 89) of identified variants were classified with “drug response” significance in ClinVar. In contrast, 98.5% (n = 6,262) of variants were listed as having either no defined or “undefined” significance in ClinVar. The remaining variants were indicated as likely benign (0.3%; n = 17), benign (0.03%; n = 2), and other (0.2%; n = 14) in ClinVar. A total of 11 *CYP2D6* variants with “drug response” significance had individual allele frequencies >0.10 (shown in Table 1) and 60 *CYP2D6* variants with “undefined” significance had individual allele frequencies >0.10 (shown in Table 2).

**TABLE 1:** Summary of allele frequencies for *CYP2D6* variants assigned “drug response” significance in ClinVar (as indicated in the *All of Us* Data Browser; last updated February 2023 and searched in October 2024; a total of 89 out of 6,359 [1.4%] variants; variants with allele frequency ≥0.10 in the overall *All of Us* population).

​	Allele frequency overall and per ancestral category								
Variant ID	RS number	Overall	African	East Asian	European	Latin American*	Middle Eastern	South Asian	Other*
22-42126611-C-G	rs1135840	0.569155	0.643879	0.696859**	0.564806	0.459859	0.622449	0.561018	0.58435
22-42127941-G-A	rs16947	0.377418	0.522755**	0.147941	0.343221	0.318624	0.479592**	0.400086	0.381486
22-42127209-T-C	rs2004511	0.200763	0.13297	0.561855**	0.227836	0.149212	0.149485	0.165296	0.213165
22-42128694-T-C	rs2267447	0.192665	0.127265	0.555369**	0.220232	0.141433	0.143959	0.161207	0.20376
22-42126390-G-A	rs28371738	0.19108	0.120567	0.528027**	0.221651	0.141335	0.141582	0.159561	0.200489
22-42132217-G-A	rs28588594	0.190967	0.120269	0.554593**	0.22049	0.140363	0.143223	0.163217	0.201803
22-42130692-G-A	rs1065852	0.190659	0.120047	0.547606**	0.220477	0.140214	0.142857	0.161283	0.201099
22-42131791-C-T	rs1080989	0.190574	0.119919	0.547521**	0.220328	0.140277	0.142857	0.161499	0.201226
22-42128945-C-T	rs3892097	0.15497	0.077924	0.006056	0.204054	0.125176	0.116368	0.104095	0.149222
22-42129809-T-C	rs3892097	0.134322	0.037776	0.002982	0.192626	0.098207	0.110256	0.085308	0.132813
22-42129819-G-T	rs28371703	0.133858	0.037591	0.002888	0.192304	0.097035	0.109974	0.085093	0.132002

*The term “Latin American” is categorized as “Americas,” and the term “Other” is categorized as “Remaining” (not belonging to one of the other ancestries or is a balanced admixture) within the Data Browser as of 15 July 2025.

**Allele frequency difference of ≥0.10 between an ancestry subpopulation *versus* the overall subpopulation.

**TABLE 2:** Summary of allele frequencies per ancestral category for *CYP2D6* variants assigned “undefined” significance in ClinVar as indicated in the *All of Us* Data Browser (last updated in February 2023 and searched in October 2024; a total of 6,262 out of 6,359 [98.5%] variants; variants with allele frequency ≥0.10 in the overall *All of Us* population).

​	Allele frequency overall and per ancestral category								
Variant ID	RS number	Overall	African	East Asian	European	Latin American*	Middle Eastern	South Asian	Other*
22-42124425-GC-G	rs146364112	0.999853	0.999741	0.999879	0.999859	0.999854	1	1	1
22-42121985-C-G	rs6002626	0.630385	0.916362**	0.673828	0.554761	0.482042	0.653944	0.549226	0.641086
22-42132027-C-T	rs139702605	0.621885	0.666726	0.718694**	0.622119	0.534851	0.663462	0.631887	0.630959
22-42130482-C-A	rs28371699***	0.594349	0.745734**	0.696665**	0.56475	0.469289	0.635204	0.564627	0.604768
22-42121632-C-G	rs5758587	0.583091	0.729463**	0.674014	0.553848	0.463014	0.632316	0.549226	0.598455
22-42134820-T-G	rs28579115	0.580161	0.689455**	0.698453**	0.564345	0.4641	0.608418	0.567399	0.591667
22-42122378-A-G	rs5758589	0.576616	0.705589**	0.681395**	0.553211	0.459154	0.620865	0.549011	0.591818
22-42127407-T-G	rs1985842***	0.572727	0.653251	0.71195**	0.566252	0.461802	0.622449	0.564119	0.587718
22-42135201-T-C	rs28670611	0.569587	0.644346	0.697373**	0.565342	0.459806	0.622449	0.566782	0.58359
22-42126069-A-C	rs35028622	0.569164	0.644322	0.698043**	0.564505	0.460297	0.622449	0.5618	0.583183
22-42135058-A-AAAC	rs141606817	0.568553	0.643824	0.697187**	0.56378	0.459465	0.622762	0.561638	0.583629
22-42134089-AT-A	rs34439031	0.56763	0.640769	0.698117**	0.563486	0.459086	0.621173	0.567026	0.581977
22-42132851-T-C	rs28439297	0.566975	0.637242	0.697726**	0.563856	0.458878	0.621173	0.56592	0.581553
22-42132844-C-A	rs28680494	0.566853	0.636964	0.697466**	0.563726	0.458885	0.62532	0.565891	0.581665
22-42133400-T-G	rs28542726	0.56676	0.63755	0.699366**	0.563553	0.458094	0.623724	0.567212	0.5809
22-42125924-A-G	rs34385013	0.565621	0.642797	0.697837**	0.560937	0.4533	0.620205	0.561154	0.580129
22-42129130-C-G	rs1058164***	0.565598	0.637682	0.695863**	0.561423	0.458332	0.621173	0.567614	0.580204
22-42130047-G-C	rs28371701***	0.466265	0.381693	0.15476	0.535137	0.394679	0.561856**	0.489822	0.474709
22-42123461-A-AT	rs367861743	0.399539	0.547068**	0.172492	0.359728	0.351072	0.533505**	0.430489	0.409407
22-42131531-G-A	rs28624811***	0.376051	0.516805**	0.150475	0.343226	0.317963	0.479592**	0.405295	0.38041
22-42123226-C-T	rs6002629	0.36987	0.508605**	0.146884	0.336977	0.314972	0.478372**	0.391445	0.37653
22-42123219-C-T	rs6002628	0.369781	0.508055**	0.146884	0.336978	0.314984	0.478372**	0.391613	0.37643
22-42123300-A-G	rs6002632	0.369532	0.504572**	0.147295	0.337366	0.315311	0.478372**	0.39152	0.376581
22-42123211-T-C	rs6002627	0.369491	0.506147**	0.146938	0.337012	0.314986	0.478372**	0.391445	0.376388
22-42123236-T-C	rs79422943	0.369329	0.50501**	0.147322	0.337013	0.314976	0.478372**	0.391398	0.376337
22-42123244-A-G	rs75305438	0.369288	0.504626**	0.147388	0.337027	0.315009	0.478372**	0.391445	0.376454
22-42123254-A-G	rs6002631	0.369277	0.504608**	0.146949	0.337051	0.31506	0.478372**	0.391445	0.376388
22-42123243-C-T	rs77989876	0.369237	0.504589**	0.147443	0.336967	0.314976	0.478372**	0.391398	0.376312
22-42123237-G-A	rs574263560	0.369236	0.504785**	0.14735	0.336921	0.314951	0.477099**	0.391398	0.376296
22-42123239-C-T	rs71328648	0.36922	0.504533**	0.147267	0.336976	0.31494	0.478372**	0.391398	0.376312
22-42123252-G-T	rs6002630	0.36921	0.504505**	0.146977	0.336989	0.314989	0.478372**	0.391445	0.376337
22-42122422-G-A	rs764481	0.369184	0.504495**	0.146949	0.337033	0.314704	0.478372**	0.391445	0.376354
22-42123312-A-G	rs201785814	0.368997	0.501856**	0.147322	0.337513	0.315346	0.477099**	0.392334	0.376323
22-42123308-G-A	rs71328649	0.367634	0.498173**	0.147316	0.33699	0.314309	0.477099**	0.391304	0.374824
22-42123311-C-T	rs190494165	0.363745	0.496643**	0.146664	0.332546	0.310983	0.451654	0.388674	0.364926
22-42127207-C-T	rs28371730***	0.35111	0.420867	0.14753	0.341668	0.308087	0.469388**	0.398879	0.358846
22-42132025-T-TCC	rs1297637179	0.348317	0.385728	0.585879**	0.35347	0.238542	0.314607	0.298922	0.362977
22-42133533-A-C	rs28369142	0.333467	0.334001	0.151642	0.345962	0.307102	0.469388**	0.404823	0.345339
22-42130565-A-G	rs1080998***	0.331207	0.334847	0.150345	0.343035	0.302314	0.468112**	0.405738	0.343601
22-42130547-T-C	rs1081000***	0.331191	0.334806	0.150252	0.342992	0.302417	0.468112**	0.405563	0.34361
22-42130559-T-G	rs28695233***	0.331149	0.334678	0.150252	0.34299	0.302326	0.468112**	0.405522	0.343647
22-42130578-C-G	rs1080995***	0.331118	0.334684	0.150261	0.342931	0.302318	0.468112**	0.40621	0.343587
22-42130560-C-G	rs29001518***	0.331095	0.334638	0.150252	0.342936	0.302278	0.468112**	0.405347	0.34352
22-42130569-G-C	rs1080997***	0.331085	0.334669	0.150289	0.342918	0.302248	0.468112**	0.405388	0.343501
22-42130571-G-T	rs1080996***	0.33108	0.334609	0.150289	0.342928	0.302245	0.468112**	0.405563	0.34353
22-42126132-CTGT-C	rs71184866	0.305477	0.24016	0.148369	0.339772	0.292496	0.436224**	0.398966	0.319128
22-42131469-C-T	rs28633410***	0.283522	0.140178	0.154326	0.344035	0.286011	0.415144**	0.408141**	0.297877
22-42132561-C-T	rs1080983	0.278828	0.137825	0.14918	0.337833	0.282712	0.409207**	0.402844**	0.293148
22-42126310-C-T	rs12169962***	0.277736	0.137261	0.146505	0.336817	0.281079	0.406888**	0.397458**	0.291951
22-42121685-A-G	rs6002625	0.275347	0.141473	0.146791	0.331465	0.27911	0.408397**	0.38951**	0.291229
22-42133212-A-G	rs28568508	0.238701	0.30953	0.547699**	0.222753	0.156699	0.174745	0.162284	0.242516
22-42132024-A-ATC​CTT	rs1292475757	0.236283	0.135695	0.220135	0.270822	0.251175	0.312857	0.271883	0.237579
22-42132969-C-T	rs28360521	0.215068	0.218345	0.547233**	0.220471	0.149664	0.151786	0.162786	0.222122
22-42125980-T-C	rs4078247	0.191925	0.121136	0.549115**	0.221798	0.141607	0.141944	0.161714	0.202277
22-42133314-C-T	rs28566059	0.190771	0.119651	0.548555**	0.220679	0.140395	0.142857	0.162354	0.201293
22-42121918-T-A	rs5751222	0.185621	0.113609	0.526879**	0.215925	0.137419	0.139949	0.156277	0.197139
22-42123238-C-T	rs5758593	0.185477	0.113354	0.525498**	0.215853	0.13737	0.139949	0.156344	0.196965
22-42132024-A-ATC​CT	rs1292475757	0.14451	0.239893	0.06837	0.127311	0.081836	0.19	0.154768	0.154973
22-42129796-G-C	rs28371705	0.134468	0.037701	0.004287	0.192982	0.097807	0.110969	0.085702	0.133211
22-42125620-GGG​GTG​GGG​AA-G	rs536156813	0.102257	0.03939	0.009302	0.134939	0.076412	0.088542	0.024459	0.122242

**Allele frequency difference of ≥0.10 between an ancestry subpopulation *versus* the overall subpopulation.

***Contains at least one sub-allele at the “definitive” allele evidence level in PharmVar: https://www.pharmvar.org/gene/CYP2D6.

In the *All of Us* population, *CYP2D6* variants with the overall highest allele frequencies and with “drug response” significance in ClinVar (majority allele) were 22-42126611-C-G (rs1135840) and 22-42127941-G-A (rs16947). *CYP2D6* variants with the overall highest allele frequencies and with “undefined” significance in ClinVar were 22-42124425-GC-G (rs146364112), 22-42121985-C-G (rs6002626), and 22-42132027-C-T (rs139702605).

#### CYP2D6 variants with ClinVar “drug response” significance by ancestry

The highest proportion of alleles classified in ClinVar as “drug response” and occurring at a frequency difference of ≥0.10 compared with the overall population (total of 11 alleles) was observed in subgroups of East Asian ancestry (7 of 11, 64% of variants; 22-42126611-C-G, rs1135840; 22-42127209-T-C, rs2004511; 22-42128694-T-C, rs2267447; 22-42126390-G-A, rs28371738; 22-42132217-G-A, rs28588594; 22-42130692-G-A, rs1065852; and 22-42131791-C-T, rs1080989; shown in Table 1). A single allele with a ClinVar “drug response” classification (1 of 11; 9%) was observed in African and Middle Eastern ancestry subgroups at a frequency difference of ≥0.10 compared with the overall population (22-42127941-G-A, rs16947). ClinVar “drug response” status conveyed for each allele was concordant with information found in PharmVar.

#### CYP2D6 variants with ClinVar “undefined” significance by ancestry

**FIGURE 1:**
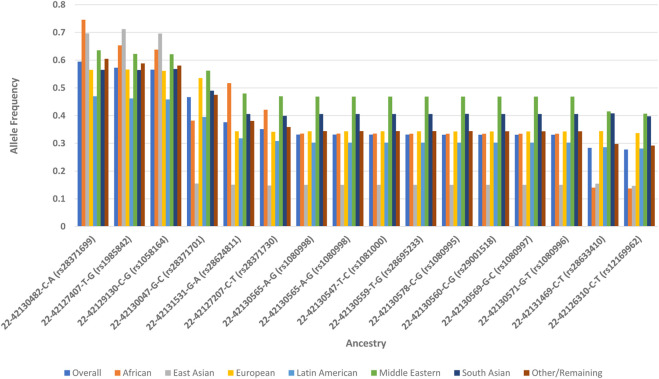
Comparison of allele frequencies for CYP2D6 alleles with ClinVar “undefined” significance, ≥0.10 frequency difference between an ancestry subgroup and the overall All of Us cohort, and ≥1 sub-allele with definitive PharmVar evidence. *Alleles were included if classified as “undefined” in ClinVar at the time of analysis. Frequency differences were calculated as the absolute difference between a given ancestry subgroup and the overall All of Us cohort, as reported in the All of Us Data Browser. “Definitive” allele evidence levels were assigned according to PharmVar allele evidence criteria.

Alleles with ClinVar “undefined” classification and a frequency difference of ≥0.10 compared with the overall population (total of 60 alleles) were observed, in descending order, in subgroups of Middle Eastern (31 of 60; 52%), African (22 of 60; 37%), East Asian (21 of 60; 35%), and South Asian (4 of 60; 7%) ancestries (shown in Table 2). Of these 60 alleles, 16 (27%) represent a range of 41–76 star allele haplotypes that contain at least one sub-allele at the “definitive” allele evidence level in PharmVar (22-42130482-C-A, rs28371699; 22-42127407-T-G, rs1985842; 22-42129130-C-G, rs1058164; 22-42130047-G-C, rs28371701; 22-42131531-G-A, rs28624811; 22-42127207-C-T, rs28371730; 22-42130565-A-G, rs1080998; 22-42130565-A-G, rs1080998; 22-42130547-T-C, rs1081000; 22-42130559-T-G, rs28695233; 22-42130578-C-G, rs1080995; 22-42130560-C-G, rs29001518; 22-42130569-G-C, rs1080997; 22-42130571-G-T, rs1080996; 22-42131469-C-T, rs28633410; and 22-42126310-C-T, rs12169962; shown in Table 3; Figure 1).

**TABLE 3:** *CYP2D6* alleles with ClinVar “undefined” significance, allele frequency difference of ≥0.10 between an ancestry subgroup and the overall All of Us cohort (as presented within the All of Us Data Browser), and at least one sub-allele at the “definitive” allele evidence level in PharmVar.

*CYP2D6* variant ID (rs number)	Ancestry subpopulation(s) (allele frequency Δ between ancestry subpopulation *versus* the overall population)	Allele name(s) with at least one sub-allele at the “definitive” allele evidence level in PharmVar^a^
22-42130482-C-A (rs28371699)	African (+0.151385) and East Asian (+0.102316)	CYP2D6*1, CYP2D6*2, CYP2D6*4, CYP2D6*10, CYP2D6*11, CYP2D6*10, CYP2D6*12, CYP2D6*14, CYP2D6*17, CYP2D6*21, CYP2D6*27, CYP2D6*28, CYP2D6*29, CYP2D6*31, CYP2D6*32, CYP2D6*35, CYP2D6*36, CYP2D6*39, CYP2D6*40, CYP2D6*41, CYP2D6*42, CYP2D6*43, CYP2D6*45, CYP2D6*46, CYP2D6*52, CYP2D6*56, CYP2D6*58, CYP2D6*59, CYP2D6*64, CYP2D6*69, CYP2D6*73, CYP2D6*84, CYP2D6*85, CYP2D6*99, CYP2D6*101, CYP2D6*111, CYP2D6*117, CYP2D6*121, CYP2D6*123, CYP2D6*125, CYP2D6*126, CYP2D6*128, CYP2D6*129, CYP2D6*132, CYP2D6*133, CYP2D6*135, CYP2D6*136, CYP2D6*138, CYP2D6*141, CYP2D6*142, CYP2D6*144, CYP2D6*145, CYP2D6*146, CYP2D6*148, CYP2D6*150, CYP2D6*152, CYP2D6*153, CYP2D6*154, CYP2D6*156, CYP2D6*157, CYP2D6*158, CYP2D6*159, CYP2D6*160, CYP2D6*161, CYP2D6*162, CYP2D6*164, CYP2D6*165, CYP2D6*166, CYP2D6*171, CYP2D6*172, CYP2D6*175, CYP2D6*177, CYP2D6*179, CYP2D6*182, CYP2D6*183, and CYP2D6*184
22-42127407-T-G (rs1985842)	East Asian (+0.139223)	CYP2D6*1, CYP2D6*2, CYP2D6*4, CYP2D6*10, CYP2D6*11, CYP2D6*12, CYP2D6*14, CYP2D6*17, CYP2D6*21, CYP2D6*28, CYP2D6*29, CYP2D6*31, CYP2D6*32, CYP2D6*35, CYP2D6*36, CYP2D6*39, CYP2D6*40, CYP2D6*41, CYP2D6*42, CYP2D6*45, CYP2D6*46, CYP2D6*52, CYP2D6*56, CYP2D6*58, CYP2D6*59, CYP2D6*64, CYP2D6*69, CYP2D6*73, CYP2D6*84, CYP2D6*85, CYP2D6*100, CYP2D6*117, CYP2D6*119, CYP2D6*121, CYP2D6*123, CYP2D6*125, CYP2D6*126, CYP2D6*127, CYP2D6*129, CYP2D6*132, CYP2D6*133, CYP2D6*135, CYP2D6*136, CYP2D6*138, CYP2D6*141, CYP2D6*142, CYP2D6*146, CYP2D6*147, CYP2D6*148, CYP2D6*149, CYP2D6*150, CYP2D6*154, CYP2D6*155, CYP2D6*156, CYP2D6*157, CYP2D6*158, CYP2D6*159, CYP2D6*161, CYP2D6*162, CYP2D6*164, CYP2D6*165, CYP2D6*166, CYP2D6*171, CYP2D6*172, CYP2D6*175, CYP2D6*177, CYP2D6*179, CYP2D6*182, CYP2D6*183, and CYP2D6*184
22-42129130-C-G (rs1058164)	East Asian (+0.130265)	CYP2D6*1, CYP2D6*2, CYP2D6*4, CYP2D6*8, CYP2D6*10, CYP2D6*11, CYP2D6*14, CYP2D6*17, CYP2D6*21, CYP2D6*28, CYP2D6*29, CYP2D6*31, CYP2D6*32, CYP2D6*35, CYP2D6*36, CYP2D6*39, CYP2D6*40, CYP2D6*41, CYP2D6*42, CYP2D6*45, CYP2D6*46, CYP2D6*49, CYP2D6*51, CYP2D6*52, CYP2D6*56, CYP2D6*58, CYP2D6*59, CYP2D6*64, CYP2D6*69, CYP2D6*73, CYP2D6*84, CYP2D6*85, CYP2D6*100, CYP2D6*117, CYP2D6*121, CYP2D6*123, CYP2D6*125, CYP2D6*126, CYP2D6*128, CYP2D6*129, CYP2D6*132, CYP2D6*133, CYP2D6*135, CYP2D6*136, CYP2D6*138, CYP2D6*141, CYP2D6*142, CYP2D6*146, CYP2D6*148, CYP2D6*149, CYP2D6*150, CYP2D6*154, CYP2D6*155, CYP2D6*156, CYP2D6*157, CYP2D6*158, CYP2D6*159, CYP2D6*161, CYP2D6*162, CYP2D6*163, CYP2D6*164, CYP2D6*165, CYP2D6*166, CYP2D6*171, CYP2D6*172, CYP2D6*175, CYP2D6*177, CYP2D6*179, CYP2D6*182, CYP2D6*183, and CYP2D6*184
22-42130047-G-C (rs28371701)	Middle Eastern (+0.095591)	CYP2D6*1, CYP2D6*2, CYP2D6*4, CYP2D6*10, CYP2D6*11, CYP2D6*12, CYP2D6*14, CYP2D6*17, CYP2D6*21, CYP2D6*28, CYP2D6*29, CYP2D6*31, CYP2D6*32, CYP2D6*35, CYP2D6*39, CYP2D6*41, CYP2D6*42, CYP2D6*45, CYP2D6*46, CYP2D6*59, CYP2D6*69, CYP2D6*73, CYP2D6*84, CYP2D6*85, CYP2D6*117, CYP2D6*121, CYP2D6*123, CYP2D6*125, CYP2D6*126, CYP2D6*128, CYP2D6*129, CYP2D6*133, CYP2D6*135, CYP2D6*136, CYP2D6*138, CYP2D6*144, CYP2D6*146, CYP2D6*148, CYP2D6*149, CYP2D6*150, CYP2D6*155, CYP2D6*156, CYP2D6*157, CYP2D6*158, CYP2D6*159, CYP2D6*161, CYP2D6*162, CYP2D6*163, CYP2D6*164, CYP2D6*165, CYP2D6*166, CYP2D6*171, CYP2D6*172, CYP2D6*175, CYP2D6*179, CYP2D6*182, CYP2D6*183, and CYP2D6*184
22-42131531-G-A (rs28624811)	African (+0.147529) and Middle Eastern (+0.133966)	CYP2D6*1, CYP2D6*2, CYP2D6*11, CYP2D6*14, CYP2D6*17, CYP2D6*21, CYP2D6*28, CYP2D6*29, CYP2D6*31, CYP2D6*32, CYP2D6*35, CYP2D6*39, CYP2D6*40, CYP2D6*41, CYP2D6*42, CYP2D6*45, CYP2D6*46, CYP2D6*51, CYP2D6*56, CYP2D6*58, CYP2D6*59, CYP2D6*73, CYP2D6*84, CYP2D6*85, CYP2D6*111, CYP2D6*117, CYP2D6*121, CYP2D6*123, CYP2D6*125, CYP2D6*126, CYP2D6*128, CYP2D6*129, CYP2D6*133, CYP2D6*135, CYP2D6*136, CYP2D6*138, CYP2D6*141, CYP2D6*144, CYP2D6*146, CYP2D6*149, CYP2D6*150, CYP2D6*154, CYP2D6*155, CYP2D6*156, CYP2D6*157, CYP2D6*158, CYP2D6*159, CYP2D6*160, CYP2D6*162, CYP2D6*163, CYP2D6*165, CYP2D6*166, CYP2D6*171, CYP2D6*172, CYP2D6*175, CYP2D6*179, CYP2D6*182, CYP2D6*183, and CYP2D6*184
22-42127207-C-T (rs28371730)	Middle Eastern (+0.118278)	CYP2D6*1, CYP2D6*2, CYP2D6*11, CYP2D6*12, CYP2D6*14, CYP2D6*17, CYP2D6*21, CYP2D6*28, CYP2D6*31, CYP2D6*32, CYP2D6*35, CYP2D6*39, CYP2D6*40, CYP2D6*41, CYP2D6*42, CYP2D6*45, CYP2D6*46, CYP2D6*56, CYP2D6*58, CYP2D6*59, CYP2D6*69, CYP2D6*73, CYP2D6*84, CYP2D6*85, CYP2D6*111, CYP2D6*117, CYP2D6*121, CYP2D6*123, CYP2D6*125, CYP2D6*126, CYP2D6*128, CYP2D6*129, CYP2D6*133, CYP2D6*135, CYP2D6*136, CYP2D6*138, CYP2D6*146, CYP2D6*148, CYP2D6*150, CYP2D6*154, CYP2D6*158, CYP2D6*159, CYP2D6*161, CYP2D6*162, CYP2D6*163, CYP2D6*166, CYP2D6*172, CYP2D6*175, CYP2D6*179, CYP2D6*182, CYP2D6*183, and CYP2D6*184
22-42130565-A-G (rs1080998)	Middle Eastern (+0.136905)	CYP2D6*1, CYP2D6*2, CYP2D6*11, CYP2D6*14, CYP2D6*15, CYP2D6*17, CYP2D6*21, CYP2D6*28, CYP2D6*31, CYP2D6*32, CYP2D6*35, CYP2D6*39, CYP2D6*40, CYP2D6*41, CYP2D6*42, CYP2D6*51, CYP2D6*56, CYP2D6*58, CYP2D6*59, CYP2D6*73, CYP2D6*84, CYP2D6*117, CYP2D6*121, CYP2D6*123, CYP2D6*126, CYP2D6*128, CYP2D6*129, CYP2D6*133, CYP2D6*135, CYP2D6*136, CYP2D6*138, CYP2D6*141, CYP2D6*144, CYP2D6*146, CYP2D6*148, CYP2D6*150, CYP2D6*154, CYP2D6*158, CYP2D6*159, CYP2D6*161, CYP2D6*162, CYP2D6*166, CYP2D6*172, CYP2D6*175, CYP2D6*179, CYP2D6*182, CYP2D6*183, and CYP2D6*184
22-42130565-A-G (rs1080998)	Middle Eastern (+0.136905)	CYP2D6*1, CYP2D6*2, CYP2D6*11, CYP2D6*14, CYP2D6*15, CYP2D6*17, CYP2D6*21, CYP2D6*28, CYP2D6*31, CYP2D6*32, CYP2D6*35, CYP2D6*39, CYP2D6*40, CYP2D6*41, CYP2D6*42, CYP2D6*51, CYP2D6*56, CYP2D6*58, CYP2D6*59, CYP2D6*73, CYP2D6*84, CYP2D6*117, CYP2D6*121, CYP2D6*123, CYP2D6*126, CYP2D6*128, CYP2D6*129, CYP2D6*133, CYP2D6*135, CYP2D6*136, CYP2D6*138, CYP2D6*141, CYP2D6*144, CYP2D6*146, CYP2D6*148, CYP2D6*150, CYP2D6*154, CYP2D6*158, CYP2D6*159, CYP2D6*161, CYP2D6*162, CYP2D6*166, CYP2D6*172, CYP2D6*175, CYP2D6*179, CYP2D6*182, CYP2D6*183, and CYP2D6*184
22-42130547-T-C (rs1081000)	Middle Eastern (+0.136921)	CYP2D6*1, CYP2D6*2, CYP2D6*11, CYP2D6*14, CYP2D6*15, CYP2D6*17, CYP2D6*21, CYP2D6*28, CYP2D6*31, CYP2D6*32, CYP2D6*35, CYP2D6*39, CYP2D6*40, CYP2D6*41, CYP2D6*42, CYP2D6*51, CYP2D6*56, CYP2D6*58, CYP2D6*59, CYP2D6*73, CYP2D6*84, CYP2D6*117, CYP2D6*121, CYP2D6*123, CYP2D6*126, CYP2D6*128, CYP2D6*129, CYP2D6*133, CYP2D6*135, CYP2D6*136, CYP2D6*138, CYP2D6*141, CYP2D6*144, CYP2D6*146, CYP2D6*148, CYP2D6*150, CYP2D6*154, CYP2D6*158, CYP2D6*159, CYP2D6*161, CYP2D6*162, CYP2D6*166, CYP2D6*172, CYP2D6*175, CYP2D6*179, CYP2D6*182, CYP2D6*183, and CYP2D6*184
22-42130559-T-G (rs28695233)	Middle Eastern (+0.136963)	CYP2D6*1, CYP2D6*2, CYP2D6*11, CYP2D6*14, CYP2D6*15, CYP2D6*17, CYP2D6*21, CYP2D6*28, CYP2D6*31, CYP2D6*32, CYP2D6*35, CYP2D6*39, CYP2D6*40, CYP2D6*41, CYP2D6*42, CYP2D6*51, CYP2D6*56, CYP2D6*58, CYP2D6*59, CYP2D6*73, CYP2D6*84, CYP2D6*117, CYP2D6*121, CYP2D6*123, CYP2D6*126, CYP2D6*128, CYP2D6*129, CYP2D6*133, CYP2D6*135, CYP2D6*136, CYP2D6*138, CYP2D6*141, CYP2D6*144, CYP2D6*146, CYP2D6*148, CYP2D6*150, CYP2D6*154, CYP2D6*158, CYP2D6*159, CYP2D6*161, CYP2D6*162, CYP2D6*166, CYP2D6*172, CYP2D6*175, CYP2D6*179, CYP2D6*182, CYP2D6*183, and CYP2D6*184
22-42130578-C-G (rs1080995)	Middle Eastern (+0.136994)	CYP2D6*1, CYP2D6*2, CYP2D6*11, CYP2D6*14, CYP2D6*15, CYP2D6*17, CYP2D6*21, CYP2D6*28, CYP2D6*31, CYP2D6*32, CYP2D6*35, CYP2D6*39, CYP2D6*40, CYP2D6*41, CYP2D6*42, CYP2D6*51, CYP2D6*56, CYP2D6*58, CYP2D6*59, CYP2D6*73, CYP2D6*84, CYP2D6*117, CYP2D6*121, CYP2D6*123, CYP2D6*126, CYP2D6*128, CYP2D6*129, CYP2D6*133, CYP2D6*135, CYP2D6*136, CYP2D6*138, CYP2D6*141, CYP2D6*144, CYP2D6*146, CYP2D6*148, CYP2D6*150, CYP2D6*154, CYP2D6*158, CYP2D6*159, CYP2D6*161, CYP2D6*162, CYP2D6*166, CYP2D6*172, CYP2D6*175, CYP2D6*179, CYP2D6*182, CYP2D6*183, and CYP2D6*184
22-42130560-C-G (rs29001518)	Middle Eastern (+0.1370025)	CYP2D6*1, CYP2D6*2, CYP2D6*11, CYP2D6*14, CYP2D6*15, CYP2D6*17, CYP2D6*21, CYP2D6*28, CYP2D6*31, CYP2D6*32, CYP2D6*35, CYP2D6*39, CYP2D6*40, CYP2D6*41, CYP2D6*42, CYP2D6*51, CYP2D6*56, CYP2D6*58, CYP2D6*59, CYP2D6*73, CYP2D6*84, CYP2D6*117, CYP2D6*121, CYP2D6*123, CYP2D6*126, CYP2D6*128, CYP2D6*129, CYP2D6*133, CYP2D6*135, CYP2D6*136, CYP2D6*138, CYP2D6*141, CYP2D6*144, CYP2D6*146, CYP2D6*148, CYP2D6*150, CYP2D6*154, CYP2D6*158, CYP2D6*159, CYP2D6*161, CYP2D6*162, CYP2D6*166, CYP2D6*172, CYP2D6*175, CYP2D6*179, CYP2D6*182, CYP2D6*183, and CYP2D6*184
22-42130569-G-C (rs1080997)	Middle Eastern (+0.137027)	CYP2D6*1, CYP2D6*2, CYP2D6*11, CYP2D6*14, CYP2D6*15, CYP2D6*17, CYP2D6*21, CYP2D6*28, CYP2D6*31, CYP2D6*32, CYP2D6*35, CYP2D6*39, CYP2D6*40, CYP2D6*41, CYP2D6*42, CYP2D6*51, CYP2D6*56, CYP2D6*58, CYP2D6*59, CYP2D6*73, CYP2D6*84, CYP2D6*117, CYP2D6*121, CYP2D6*123, CYP2D6*126, CYP2D6*128, CYP2D6*129, CYP2D6*133, CYP2D6*135, CYP2D6*136, CYP2D6*138, CYP2D6*141, CYP2D6*144, CYP2D6*146, CYP2D6*148, CYP2D6*150, CYP2D6*154, CYP2D6*158, CYP2D6*159, CYP2D6*161, CYP2D6*162, CYP2D6*166, CYP2D6*172, CYP2D6*175, CYP2D6*179, CYP2D6*182, CYP2D6*183, and CYP2D6*184
22-42130571-G-T (rs1080996)	Middle Eastern (+0.137032)	CYP2D6*1, CYP2D6*2, CYP2D6*11, CYP2D6*14, CYP2D6*15, CYP2D6*17, CYP2D6*21, CYP2D6*28, CYP2D6*31, CYP2D6*32, CYP2D6*35, CYP2D6*39, CYP2D6*40, CYP2D6*41, CYP2D6*42, CYP2D6*51, CYP2D6*56, CYP2D6*58, CYP2D6*59, CYP2D6*73, CYP2D6*84, CYP2D6*117, CYP2D6*121, CYP2D6*123, CYP2D6*126, CYP2D6*128, CYP2D6*129, CYP2D6*133, CYP2D6*135, CYP2D6*136, CYP2D6*138, CYP2D6*141, CYP2D6*144, CYP2D6*146, CYP2D6*148, CYP2D6*150, CYP2D6*154, CYP2D6*158, CYP2D6*159, CYP2D6*161, CYP2D6*162, CYP2D6*166, CYP2D6*172, CYP2D6*175, CYP2D6*179, CYP2D6*182, CYP2D6*183, and CYP2D6*184
22-42131469-C-T (rs28633410)	Middle Eastern (+0.131622) and South Asian (+0.124619)	CYP2D6*1, CYP2D6*2, CYP2D6*11, CYP2D6*14, CYP2D6*21, CYP2D6*28, CYP2D6*31, CYP2D6*32, CYP2D6*35, CYP2D6*39, CYP2D6*41, CYP2D6*42, CYP2D6*51, CYP2D6*56, CYP2D6*59, CYP2D6*84, CYP2D6*111, CYP2D6*117, CYP2D6*121, CYP2D6*123, CYP2D6*126, CYP2D6*128, CYP2D6*129, CYP2D6*133, CYP2D6*135, CYP2D6*136, CYP2D6*138, CYP2D6*144, CYP2D6*146, CYP2D6*148, CYP2D6*158, CYP2D6*159, CYP2D6*161, CYP2D6*162, CYP2D6*166, CYP2D6*172, CYP2D6*175, CYP2D6*179, CYP2D6*182, CYP2D6*183, and CYP2D6*184
22-42126310-C-T (rs12169962)	Middle Eastern (+0.123366) and South Asian (+0.113936)	CYP2D6*1, CYP2D6*2, CYP2D6*11, CYP2D6*14, CYP2D6*21, CYP2D6*28, CYP2D6*31, CYP2D6*32, CYP2D6*35, CYP2D6*39, CYP2D6*41, CYP2D6*42, CYP2D6*59, CYP2D6*69, CYP2D6*84, CYP2D6*111, CYP2D6*117, CYP2D6*119, CYP2D6*121, CYP2D6*123, CYP2D6*126, CYP2D6*128, CYP2D6*129, CYP2D6*133, CYP2D6*135, CYP2D6*136, CYP2D6*138, CYP2D6*146, CYP2D6*148, CYP2D6*150, CYP2D6*158, CYP2D6*159, CYP2D6*161, CYP2D6*162, CYP2D6*166, CYP2D6*172, CYP2D6*175, CYP2D6*179, CYP2D6*182, CYP2D6*183, and CYP2D6*184

^a^ https://www.pharmvar.org/gene/CYP2D6.

## Discussion

In this study, we observe that the majority of *CYP2D6* alleles present among the *All of Us* cohort (98.5%; n = 6,262) remain undefined or unclassified in ClinVar, which is consistent with current *All of Us* reports indicating that the program has identified more than 275 million previously unreported genetic variants (The All of Us Research Program Genomics Investigators et al., 2024). Moreover, only 1.4% (n = 89) of identified *CYP2D6* alleles among the *All of Us* cohort were classified as having “drug response” significance in ClinVar. Among these, only two (2) *CYP2D6* alleles were observed at very high frequencies in the overall *All of Us* cohort (thus, “majority alleles”; 22-42126611-C-G [rs1135840] and 22-42127941-G-A [rs16947]). The highest proportion of alleles with a “drug response” significance classification in ClinVar and a frequency difference of ≥0.10 compared with the overall population was observed in subgroups of East Asian ancestry. This finding suggests a testable hypothesis that subgroups of East Asian ancestry or descent are more likely than the general population to carry *CYP2D6* alleles of established therapeutic relevance, which might measurably improve therapeutic outcomes when PGx testing is implemented in this subgroup.

On the other hand, *CYP2D6* alleles with the ClinVar “undefined” classification and frequency differences ≥0.10 compared with the overall population were frequently observed (in descending order of difference) in subgroups of Middle Eastern, African, East Asian, and South Asian ancestries. This finding suggests another testable hypothesis that subgroups of Middle Eastern, African, East Asian, or South Asian ancestries or descent are more likely than the general population to carry previously undiscovered *CYP2D6* alleles with pharmacological implications, which might also measurably improve therapeutic discovery and outcomes among these ancestry subgroups. This hypothesis is supported or substantiated by the fact that several (n = 16) *CYP2D6* alleles with “undefined” classification in ClinVar are haplotypes that contain at least one sub-allele at the “definitive” allele evidence level in PharmVar. This fact also suggests that those 16 alleles may warrant reclassification as “drug response” in ClinVar (and subsequently the *All of Us* Data Browser).

Our findings align with recent work showing significant odds ratios for *CYP2D6* star allele frequency differences across ancestral groups. For example, a recent study showed a higher *1 allele frequency for *CYP2D6* among individuals with self-reported non-European ancestry compared to those with self-reported European ancestry (odds ratio [OR]: 1.87; confidence interval [CI]: 1.4–2.49) (Siu et al., 2025). Another study characterizing Asian ancestry subgroups reported that Chinese and Malay populations predominantly exhibit *36 + *10, *10, and *36 alleles, while Indian populations more frequently carry *2, *4, *5, and *41 alleles (Maulana et al., 2024). These observations are consistent with our findings showing higher frequencies of multiple *CYP2D6* star alleles, including but not limited to *1, among populations of African, East Asian, Middle Eastern, and South Asian ancestries. Collectively, these results reinforce recent recommendations to expand ancestral diversity in pharmacogenomic studies to capture global *CYP2D6* genetic variation and its clinical impact on therapeutic outcomes (Kehinde et al., 2023).

Interestingly, however, our findings did not identify the CYP2D6*5 allele (whole-gene deletion variant) within the All of Us cohort. In contrast, the US Million Veteran Project reported CYP2D6*5 allele frequencies across their European, African, Native American, and Hispanic (European, African, and Native American) cohorts (Markianos et al., 2023). Relatively high copy number variations (i.e., 3+ copies) of CYP2D6*5 were observed among European (allele frequency of 14.80) and European–Hispanic (allele frequency of 16.89) subgroups compared to other subgroups (African [allele frequency of 11.96], Hispanic only [allele frequency of 11.58], African–Hispanic [allele frequency of 10.03], and Native American–Hispanic [allele frequency of 2.45]) (Markianos et al., 2023). Therefore, future work should validate whether the CYP2D6*5 allele is truly absent from the *All of Us* cohort or present at frequencies below the 0.10 threshold used in our analysis.

The ClinVar genomic variant classification convention includes but is not limited to “drug response,” although there are inconsistencies in how laboratories classify the same variant (Amendola et al., 2016). Although ClinVar is a widely used resource for lay audiences and open data users to understand the clinical significance of genomic variants, our findings suggest that ClinVar may provide only limited value for understanding nuances associated with “drug response” significance (beyond general references to the corresponding peer-reviewed literature). In the absence of pharmacological context [i.e., mechanism of action; (re)absorption, distribution, metabolism, elimination (ADME); metabolizer type such as poor, normal, intermediate, rapid, and ultrarapid] coupled with evidentiary considerations found in other public resources such as CPIC and ClinGen, relying on the Data Browser could result in confusion or misinterpretation (*versus* value) among public users, which may undermine public trust in the scientific community (Lu et al., 2020; Caudle et al., 2017; Caudle et al., 2020). To improve clarity and utility, we recommend that public-facing resources, such as the Data Browser, also include hyperlinks to pharmacological resources such as the PharmVar Consortium. PharmVar aggregates pharmacogenomic evidence, standardizes nomenclature, and summarizes structural variations and copy number variation for biomarkers such as *CYP2D6.* PharmVar also provides helpful guidance on best practices for electronic health record integration to support more accurate *CYP2D6* genotype–phenotype correlation studies and interpretations (Turner et al., 2023).

Further attempts to characterize and confirm the pharmacological significance of *CYP2D6* variants across ancestry subgroups, compared to the overall population, will require investigations across multiple databases and registries. For instance, a recent study involving more than 98,000 All of Us participants combined All of Us data with other databases (i.e., GnomAD data [https://gnomad.broadinstitute.org/downloads] and VIP data [https://gitlab.com/bcm-hgsc/neptune]) (Venner et al., 2024). This approach enabled a comparison of pathogenic variant rates between ancestral groups. Among the findings, individuals of European ancestry had the highest overall rate of variants currently reported as pathogenic in ClinVar, while other ancestry groups (African and Latino/Admixed American ancestry) showed comparatively lower rates. Although this study was not pharmacological in nature, a similar cross-database approach could be applied to validate or replicate our present findings. In addition, our observations can also be compared with those found in other similar databases [US FDA Database of Pharmacogenomic Information in Ethnic Minority Populations (dbPGxEMP), UK Biobank, Genomics England, gnomAD, and TOPMed] to confirm or strengthen the validity of our findings.

There are limitations to our study and findings that should be noted. First, Data Browser findings for *CYP2D6* are based on genetic ancestry. However, the AoU Program defines ancestry using uniform manifold approximation and projection (UMAP) representations of 16-dimensional principal component analysis from whole-genome sequencing, combined with self-described race and ethnicity. This methodology has been publicly critiqued by members of the computational biology community for its limitations in capturing ancestry-related variation (The All of Us Research Program Genomics Investigators et al., 2024; Patchter, 2024). Second, given our focus on ancestry, our present observations may be less applicable to clinical settings and policymaking where decisions are typically based on self-reported race and ethnicity. Our exclusive reliance on aggregated data from the All of Us Data Browser may also prevent individual-level analysis, which restricted our ability to draw definitive conclusions about ancestry-specific differences and clinical significance. Finally, the *All of Us* Data Browser omits copy number and structural variants that can be critical for accurately assessing the pharmacological relevance, as is the case for *CYP2D6* (Verma et al., 2022; Jarvis et al., 2019; Beoris et al., 2016; Frear and Sherman, 2026).

We recommend that our summary-level analysis of *CYP2D6* prevalence by ancestry be used solely for hypothesis generation, ideally in conjunction with deeper publicly available findings from complementary resources such as the *All of Us* All by All results map (https://allbyall.researchallofus.org/). Researchers with institutional access can also leverage the *All of Us* Researcher Workbench, a secure, cloud-based platform, to analyze *All of Us* data at the individual level. However, given personal and cultural sensitivities surrounding genomic research, researchers are especially encouraged to leverage the Workbench to validate and discuss our observations in partnership with local communities and other clinical or pharmacological experts.

Altogether, our findings highlight multiple opportunities for action moving forward. Actions can involve improving scientific communication and thus public understanding of *CYP2D6* pharmacogenomics by harmonizing ClinVar and PharmVar classifications, along with strengthening pharmacovigilance and public health safety surveillance for drugs metabolized (either partially or fully) by *CYP2D6* (see Supplementary Table 1). Additional actions may include reducing conflations between biological *versus* social factors in genomic medicine and research by reporting pharmacogenomic findings by ancestry with clarity and precision. Finally, this would enable distributional cost-effectiveness analyses in genomic medicine that explicitly accounts for ancestral subgroup variation (Thottunkal et al., 2025; Smith et al., 2025).

## Conclusion

*CYP2D6* plays a central role in pharmacogenomics and public health and offers valuable opportunities to enhance public understanding of pharmacogenetic relevance across diverse populations. These findings highlight the importance of considering ancestry-informed allele distributions in future research and policy decisions. Public-facing data resources such as the *All of Us* Data Browser hold significant potential to support precision medicine through increased public engagement, knowledge sharing, and open access to genomic insights. Our focus on improving public-tier data in the *All of Us* Data Browser is critical to facilitate broad public understanding, trust, education, and investment in nationally serving research initiatives such as *All of Us*.

## Data Availability

The original contributions presented in the study are included in the article/Supplementary Material; further inquiries can be directed to the corresponding author.
