# Supplementary material for: Opportunities to improve open All of Us data to convey CYP2D6 pharmacological relevance and interpretation overall and according to ancestry
Source: Front Pharmacol. 2026 Apr 13;17:1760362. doi: 10.3389/fphar.2026.1760362 (PMC13111366; doi:10.3389/fphar.2026.1760362)
Supplement: Supplementary file 1 [file Supplementaryfile1.pdf]

Therapeutic Area	Drug
Anesthesiology	Codeine
	Lofexidine
	Oliceridine
	Tramadol
Cardiology	Carvedilol
	Metoprolol
	Nebivolol
	Propafenone
	Propranolol
	Quinidine
Dental	Cevimeline
Gastroenterology	Metoclopramide
	Ondansetron
	Palonosetron
Gynecology	Flibanserin
Inborn Errors of Metabolism	Eliglustat
Infectious Diseases	Quinine Sulfate
Neurology	Deutetrabenazine
	Dextromethorphan and Quinidine
	Donepezil
	Galantamine
	Meclizine
	Tetrabenazine
	Valbenazine
Oncology	Gefitinib
	Rucaparib
	Tamoxifen
Psychiatry	Amitriptyline
	Amoxapine
	Amphetamine
	Aripiprazole
	Aripiprazole Lauroxil
	Atomoxetine
	Brexpiprazole
	Bupropion
	Cariprazine
	Citalopram
	Clomipramine
	Clozapine
	Desipramine
	Desvenlafaxine
	Doxepin
	Duloxetine
	Escitalopram
	Fluoxetine
	Fluvoxamine
	Iloperidone
	Imipramine
	Modafinil
	Nefazodone
	Nortriptyline
	Paliperidone
	Paroxetine
	Perphenazine
	Pimozide
	Pitolisant
	Protriptyline
	Risperidone
	Thioridazine
	Trimipramine
	Venlafaxine
	Viloxazine
	Vortioxetine
Pulmonary	Arformoterol
	Formoterol
	Umeclidinium
Rheumatology	Upadacitinib
Urology	Darifenacin
	Fesoterodine
	Mirabegron
	Tamsulosin
	Tolterodine
